# The risk of laryngitis with herpes zoster infection: A nested case-control study using data from the Korean National Sample Cohort

**DOI:** 10.1371/journal.pone.0261366

**Published:** 2021-12-10

**Authors:** Young-Hoon Joo, Hyun-Jin Lee, Jun-Ook Park, Young Joon Seo, Tae Hoon Kong, Kyoung Ho Park

**Affiliations:** 1 Department of Otolaryngology-Head and Neck Surgery, College of Medicine, The Catholic University of Korea, Seoul, Korea; 2 Department of Otorhinolaryngology, Yonsei University Wonju College of Medicine, Wonju, South Korea; University of Colorado School of Medicine, UNITED STATES

## Abstract

**Background:**

Whether herpes zoster infection (HZI) affects laryngitis incidence remains unknown.

**Objective:**

The purpose of this population-based retrospective study was to analyze the relationship between laryngitis and HZI using data from the Korean Health Insurance Review and Assessment Service—National Sample Cohort.

**Methods:**

This study analyzed 1,197,093 medical claim codes from 2018. Patients with HZI (ICD-10: B02) were retrospectively identified. Laryngeal diseases were defined by ICD-10 codes for five subgroups: 1) malignant disease, 2) benign disease, 3) vocal cord palsy, 4) inflammatory disease, and 5) reflux disease.

**Results:**

Among the Korean population older than 20 years, 12,809 experienced HZI. Subjects with HZI were more likely to be older (mean age: 51.54 years vs. 48.06 years, *p* <0.0001). The proportion of subjects with laryngeal disease was higher in those with HZI than in those without HZI (55.55% vs. 41.37%, *p* <0.0001). Laryngeal disease was significantly associated with HZI in multiple regression analysis (odds ratio (OR) = 1.77, 95% confidence interval: 1.71–1.84) after adjusting for age, sex, hypertension, diabetes, dyslipidemia, ischemic heart disease, cerebral stroke, and depression. Among laryngeal disease subgroups, inflammatory disease (OR = 1.05; 95% CI: 1.01–1.09) and reflux (OR = 1.20; 95% CI: 1.15–1.25) were associated with HZI.

**Conclusions:**

HZI is independently associated with laryngitis. Results of this study have implications for etiological investigations and prevention strategies for laryngitis.

## Introduction

Herpes zoster laryngitis is a rare manifestation of herpes zoster infection (HZI), which is widely described in case reports. HZI is caused by reactivation of latent varicella-zoster virus [1]. In South Korea in 2015, there were 469,268 HZI cases, and the prevalence was 9.22/1000 person-years [2]. Laryngitis occurs when the larynx become inflamed, swollen, and irritated. Acute laryngitis is inflammation of the vocal fold mucosa and larynx that lasts less than three weeks [3, 4]. Chronic laryngitis is diagnosed when signs and symptoms last longer than three weeks and can have infectious or non-infectious causes [4–6]. In Korea, the prevalence of chronic laryngitis was 3.8±0.7% [7].

In herpes zoster laryngitis, the varicella zoster virus affects the glossopharyngeal and vagus nerves [6]. A deterministic feature for herpes zoster laryngitis diagnosis is the presence of mucosal lesions consisting of edema and small erosions suggesting ruptured vesicles, with the lesions being confined to one half of the larynx. The sensory distribution of the vagus nerve includes the internal branch of the superior laryngeal nerve, which affects the mucosa of pharynx and larynx from the level of the epiglottis to the vocal folds [8].

Appropriate public health information should be based on best-available evidence and should contribute to proper provision of healthcare and preventive screenings. The relationship between laryngitis and HZI in adults has not been investigated in population-based studies. Thus, the objective of this study was to determine the association between HZI and laryngitis while controlling for possible confounders in a South Korean population based on the Korean Health Insurance Review and Assessment Service—National Sample Cohort (HIRA-NSC).

## Methods

### Ethics statement

This research protocol was approved by the Institutional Review Board of The Catholic University of Korea. Informed consent was waived because anonymous data were provided by the Korean National Health Insurance Service (KNHIS).

### Participant selection

This national cohort study relied on data from HIRA-NSC, a cohort of people who participated in health screening programs provided by the KNHIS in the Republic of Korea. Of 1,201,156 cases with 1,197,093 medical claim codes in 2018, we included participants who were diagnosed with HZI (ICD-10: B02). We excluded participants who were younger than 20 years (n = 4,063). The included participants were further narrowed by requiring treatment ≥2 times or antiviral medication ≥1 time. A total of 12,809 participants with HZI was selected. Laryngeal diseases were defined by the ICD-10 codes for five subgroups: 1) laryngeal malignant disease, including malignant neoplasm of the intrinsic larynx (C32), malignant neoplasm of the supraglottis (C32.1), malignant neoplasm of the subglottis (C32.2), malignant neoplasm of the laryngeal cartilage (C32.3), malignant neoplasm with overlapping lesion of the larynx (C32.8), malignant neoplasm of the larynx, unspecified lesion (C32.9), and carcinoma in situ of the larynx (D02.0); 2) laryngeal benign disease, including benign neoplasm of the larynx (D14.1), vocal cord and larynx diseases, NEC (J38), vocal cord and larynx polyps (J38.1), and vocal cord nodules (J38.2); 3) vocal cord palsy, including vocal cord and larynx paralysis (J38.0); 4) laryngeal inflammatory disease, including acute laryngitis (J04.0), acute laryngotracheitis (J04.2), acute laryngopharyngitis (J06.0), chronic laryngitis (J37.0), and chronic laryngotracheitis (J37.1); and 5) laryngeal reflux disease, including gastro-esophageal reflux disease without esophagitis (K219).

**Fig 1:**
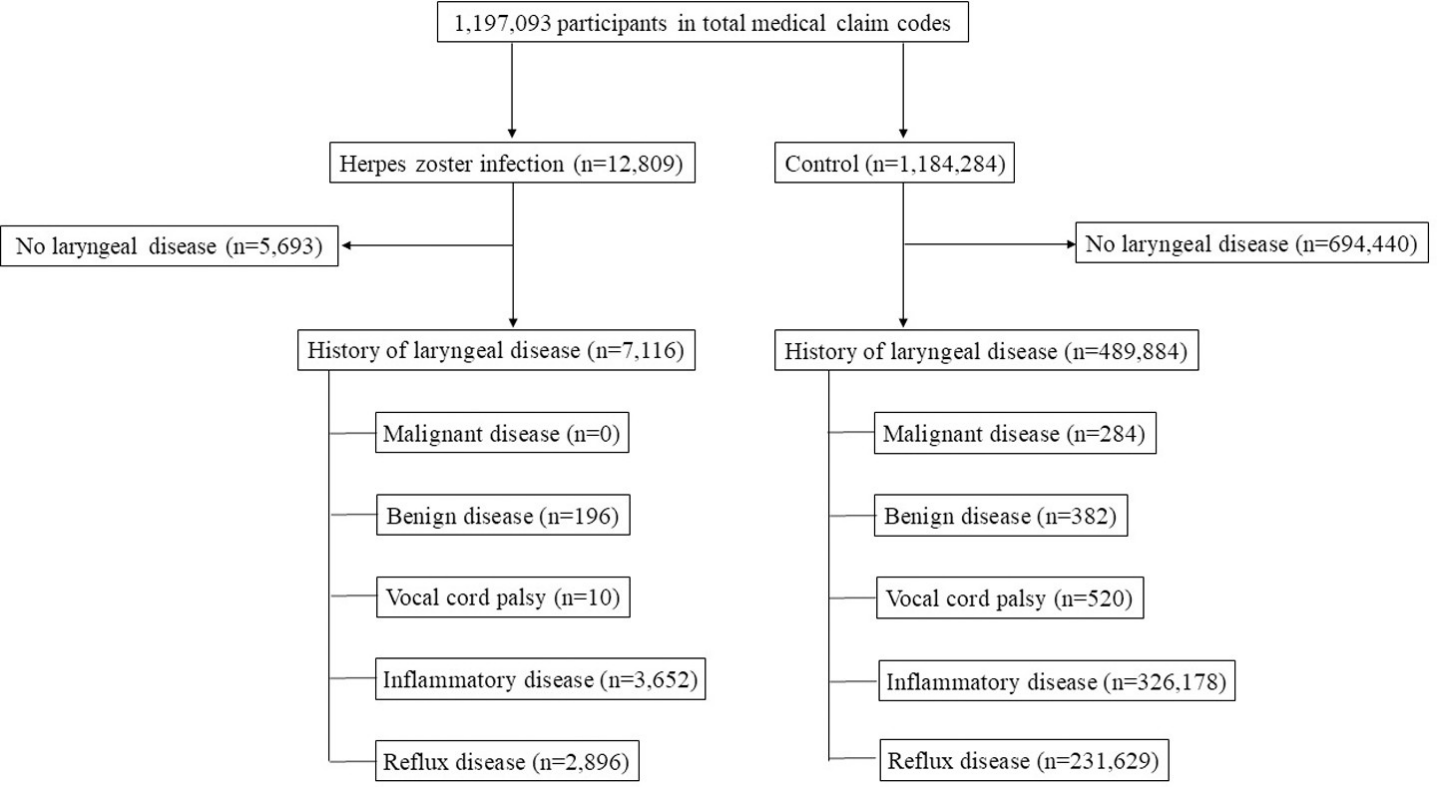
A schematic illustration of the participant selection process used for the present study.

Participants with herpes zoster were compared with cohort participants (control group) who were never diagnosed with herpes zoster. Characteristics of age, laryngeal disease group, sex, and past medical history (hypertension, diabetes, and dyslipidemia) were included. Finally, 12,809 adult participants with HZI and 1,184,284 control participants were included (Fig 1). The laryngeal disease subgroups could contain duplicate subjects because a participant could have two or more diagnoses. When comparing laryngeal disease groups, we categorized patients into the subgroup that matched their first primary diagnosis.

### Variables

Participants’ past medical histories were evaluated using ICD-10 codes. To ensure accurate diagnoses, hypertension (I10 and I15), diabetes (E10–E14), and dyslipidemia (E78) were regarded as present if a participant was treated ≥2 times. Ischemic heart disease (I24 and I25) and cerebral stroke (I60–I66) were present if the participants were treated ≥1 time. Depression was defined using ICD-10 codes F31 (bipolar affective disorder) through F39 (unspecified mood disorder) assigned by a psychiatrist.

### Statistical analyses

All statistical analyses were performed using the Statistical Analysis Software (SAS) survey procedure v. 9.3 (SAS Institute, Cary, NC, USA) to reflect the complex sampling design and sampling weights of HIRA-NSC and to provide nationally representative prevalence estimates. Prevalence and 95% confidence interval (CI) of chronic laryngitis were calculated. To test the associations between laryngeal disease and risk factors using a complex sampling design in univariate analyses, the Rao-Scott Chi-square test was performed using PROC SURVEYFREQ, and logistic regression analysis was performed using PROC SURVEYLOGISTIC. Simple and multiple linear regression analyses were performed to examine the association between laryngeal disease and herpes zoster infection. Model 1 was unadjusted. Model 2 was adjusted for age and sex. Model 3 was additionally adjusted for income, hypertension, diabetes, dyslipidemia, ischemic heart disease, cerebral stroke, and depression histories. Two-tailed analyses were conducted, and p-values <0.05 were considered to indicate significance.

## Results

### General characteristics of the study population

Study subject demographics are summarized in Table 1. Among 1,197,093 participants >20 years of age, 12,809 had HZI. The mean age of HZI patients was significantly higher than those without HZI (51.54 ± 15.02 years vs. 48.06 ± 15.92 years, *p* <0.0001). The percentage of subjects who had laryngeal disease was much higher in those with HZI than in those without HZI (55.55% vs. 41.37%, *p* <0.0001). The number of patients with laryngeal disease was 0 for malignant disease, 196 for benign disease, 10 for vocal cord palsy, 3652 for inflammatory disease, and 2896 for reflux disease. Inflammatory disease and reflux disease were significantly more frequent in the HZI groups at baseline. HZI was also significantly associated with hypertension (*p* = 0.0447), dyslipidemia (*p* <0.0001), and depression (*p* = 0.0020).

**Table 1:** Baseline characteristics of the study population (N = 1,197,093).

Parameter	Herpes zoster infection	Control	p-value
	(n = 12,809)	(n = 1,184,284)	
Sex			<0.0001*
Male	4,634 (36.18%)	574,039 (48.47%)	
Female	8,175 (63.82%)	610,245 (51.53%)	
Age	51.54 ± 15.02 years	48.06 ± 15.92 years	<0.0001*
Hypertension			
Yes	3,412 (26.64%)	220,987 (18.66%)	0.0447*
No	9,397 (73.36%)	963,297 (81.34%)	
Diabetes			
Yes	1,803 (14.08%)	110,494 (9.33%)	0.8934
No	11,005 (85.92%)	1,073,790 (90.67%)	
Dyslipidemia			
Yes	3364 (26.26%)	187,946 (15.87%)	<0.0001*
No	9445 (73.74%)	996,338 (84.13%)	
Heart disease			
Yes	220 (1.72%)	12,317 (1.04%)	0.8624
No	12,589 (98.28%)	1,171,967 (98.96%)	
Cerebral stroke			
Yes	474 (3.70%)	24,396 (2.06%)	0.6005
No	12,335 (96.30%)	1,159,888 (97.94%)	
Depression			
Yes	1,007 (7.86%)	55,780 (4.71%)	0.0020*
No	11,802 (96.30%)	1,128,504 (95.29%)	
Laryngeal disease			<0.0001*
Yes	6,754 (52.73%)	489,884 (41.37%)	
No	6,055 (47.27%)	694,400 (58.63%)	
Malignant disease			0.0796
Yes	0 (0%)	284 (0.02%)	
No	12,809 (100%)	1,184,000 (99.98%)	
Benign disease			0.7670
Yes	196 (1.53%)	382 (1.49%)	
No	12,613 (98.47%)	1,183,902 (98.51%)	
Vocal cord palsy			0.0675
Yes	10 (0.08%)	520 (0.04%)	
No	12,799 (99.92%)	1,183,764 (99.96%)	
Inflammatory disease			0.0146*
Yes	3,652 (28.51%)	326,178 (27.54%)	
No	9,157 (71.49%)	858,106 (72.46%)	
Reflux disease			<0.0001*
Yes	2,896 (22.61%)	231,629 (19.56%)	
No	9,913 (77.39%)	952,655 (80.44%)	

*Chi-square test, significance at *p* <0.05.

Multivariable-adjusted odds ratio (OR) for laryngeal disease with HZI are presented in Table 2. After adjusting for age, sex, hypertension, diabetes, dyslipidemia, ischemic heart disease, cerebral stroke, and depression, the OR for laryngeal disease was 1.77 times higher (95% CI: 1.71–1.84) in participants with HZI than in those without HZI.

**Table 2:** Crude and adjusted odd ratios (95% confidence interval) of laryngeal disease for herpes zoster infection.

Characteristics	Herpes zoster infection	Control	Odd ratio (95% confidence interval)		
	(N = 12,809)	(N = 1,184,284)	Model 1	Model 2	Model 3
Laryngeal disease			2.39(2.01, 2.85)	1.80 (1.23, 2.62)	1.77 (1.71–1.84)
Yes	6,754 (52.73%)	489,884 (41.37%)			
No	6,055 (47.27%)	694,400 (58.63%)			

Model 1: Unadjusted.

Model 2: Adjusted for age, sex

Model 3: Adjusted for age, sex, income, hypertension, diabetes, dyslipidemia, ischemic heart disease, cerebral stroke, and depression histories.

Table 3 presents data on the relationship between HZI and laryngeal disease risk according to logistic regression models. In our regression analysis, HZI was associated with laryngeal inflammatory disease (OR = 1.05; 95% CI: 1.01–1.09) and laryngeal reflux disease (OR = 1.20; 95% CI: 1.15–1.25) after adjusting for age, sex, hypertension, diabetes, dyslipidemia, ischemic heart disease, cerebral stroke, and depression histories.

**Table 3:** Subgroup analysis of crude and adjusted odd ratios (95% confidence interval) of five subgroups of laryngeal diseases for herpes zoster.

Laryngeal disease	Herpes zoster infection	Control	Odd ratio (95% confidence interval)		
	(N = 12,809)	(N = 1,184,284)	Model 1	Model 2	Model 3
Malignant disease			0.0 (0.00–1.21)	0.0 (0.00–1.21)	0.0 (0.00–1.21)
Yes	0 (0%)	284 (0.02%)			
No	12,809 (100%)	1,184,000 (99.98%)			
Benign disease			1.29 (1.08–1.53)	1.17 (1.01–1.37)	1.03 (0.86–1.22)
Yes	196 (1.53%)	382 (1.49%)			
No	12,613 (98.47%)	1,183,902 (98.51%)			
Vocal cord palsy			1.32 (1.13, 1.55)	1.39 (1.17, 1.64)	1.78 (0.85–3.30)
Yes	10 (0.08%)	520 (0.04%)			
No	12,799 (99.92%)	1,183,764 (99.96%)			
Inflammatory disease			1.25 (1.03, 1.50)	1.21 (1.00, 1.47)	1.05 (1.01–1.09)
Yes	3,652 (28.51%)	326,178 (27.54%)			
No	9,157 (71.49%)	858,106 (72.46%)			
Reflux disease			1.82 (1.26, 2.64)	1.82 (1.25, 2.64)	1.20 (1.15–1.25)
Yes	2,896 (22.61%)	231,629 (19.56%)			
No	9,913 (77.39%)	952,655 (80.44%)			

Model 1: Unadjusted.

Model 2: Adjusted for age, sex

Model 3: Adjusted for age, sex, income, hypertension, diabetes, dyslipidemia, ischemic heart disease, cerebral stroke, and depression histories.

## Discussion

Varicella-zoster virus infection causes two clinically distinct forms of disease: varicella (chickenpox) and herpes zoster (shingles). The first contact with this virus, which is transmitted by airborne viral particles released from the skin of the infected person, will result in varicella [9]. Primary infection with varicella zoster virus is characterized by vesicular lesions in different stages of development on the face, trunk, and extremities. After symptom resolution, the virus travels through sensory fibers to the dorsal fascia of the spinal cord and can remain dormant for many years. Clinically, HZI is characterized by unilateral, painful, vesicular rash with a dermatomal distribution. The most common presentation of HZI in the head and neck region is called Ramsay Hunt syndrome, which involves neurons in the geniculate ganglion of the sensory branch in the face and ears. Ramsay Hunt syndrome is defined as an acute peripheral facial neuropathy associated with erythematous vesicular rash of the skin of the ear canal, auricle, and/or mucous membrane of the oropharynx [1]. However, HZI of the larynx is rare compared with Ramsay Hunt syndrome [10–15].

In this cross-sectional study, a positive and independent relationship was observed between laryngitis and HZI. This is the first population-based study to investigate the association between laryngitis and HZI in adults. Nisa et al. performed a systematic review of pharyngolaryngeal involvement by HZI and found that (1) it appears most frequently in the context of cranial polyneuropathy, most often involving cranial nerves VII and VIII; (2) its early signs are odynophagia and dysphonia; (3) lateralization of symptoms and velar and pharyngolaryngeal hemiparesis and hemianesthesia are constant features; (4) other than clinical suspicion, the only reliable diagnostic tool is serological confirmation, as radiological findings are mostly normal; and (5) the cure rate is low (26%), with many patients having long-term neural deficits that can lead to swallowing and voice disorders [16].

Laryngitis is a common condition. Chronic laryngitis has an incidence of 3.5 new cases per 1,000 people annually [4]. It is often treated by primary care practitioners and otolaryngologists. Viruses, bacteria, and fungi or molds can infect the larynx and cause acute laryngitis. Most cases of acute laryngitis are triggered by a temporary viral infection. This diagnosis can often be obtained from a thorough history of present illness from the patient [17]. If there is no infection history or disease contact, additional causes of non-infectious laryngitis must be explored. Symptoms often include voice changes, early vocal fatigue, or dry cough. Chronic laryngitis is generally caused by exposure to irritants over time. Laryngeal examination using a fiber optic laryngoscope generally reveals diffuse edema or erythema of the laryngeal mucosa. Chronic laryngitis has diverse causes related to laryngeal irritation: (1) acid reflux, also called gastroesophageal reflux disease, (2) smoking or excessive alcohol use, and (3) inhaled irritants, such as chemical fumes, allergens, or smoke [5].

In this study, we investigated the correlation between laryngitis and HZI, and we show that HZI was closely associated with risk of laryngeal disease. Compared with cases without HZI, the risk of laryngeal disease was higher in those with HZI (OR = 1.77). Furthermore, HZI was the only risk factor associated with laryngitis (OR = 1.05 for inflammatory disease; OR = 1.20 for reflux disease) in multiple regression analysis, although it was not significantly correlated with other laryngeal diseases such as malignancy, benign disease, or vocal cord palsy. In a recent published study using the Korean National Health Insurance claims database, the risk of cancer in the larynx was significantly decreased in the HZ group compared with the non-HZ group [18]. However, Pahor reported four cases of herpes zoster laryngitis and suggested that it might be an underlying cause of many cases of idiopathic vocal cord paralysis [19]. Patients with herpes zoster laryngitis showed typical symptoms such as sore throat, voice change, and aspiration [5]. Therefore, herpes zoster laryngitis is often misdiagnosed as common viral laryngitis when HZI is not suspected. Previously, physicians often misdiagnosed patients with herpes zoster laryngitis as having common viral laryngitis before knowledge of the disease entity of herpes zoster laryngitis [20]. Although the underlying pathophysiology of this phenomenon is not clearly understood, HZI could be a component of many consequences of laryngitis. The main treatments for herpes zoster laryngitis include high-dose steroids and antiviral drugs. Several studies reported that antiviral drugs offer rapid pain relief and prevent post-herpetic neuralgia, which is more severe in older age [21, 22]. Therefore, if patients with herpes zoster-related disease have a sore throat or complain of other laryngeal symptoms, herpes zoster laryngitis should be suspected, and a laryngeal evaluation with early introduction of antiviral drugs should be performed.

The major strength of this study is that many covariates were adjusted in our models. Additionally, this was the first population-based study to estimate the association of laryngitis with HZI in the general Korean population. This study also has some limitations. First, we relied on cross-sectional data, which are not suitable for defining the relationship between laryngitis and HZI. Second, we did not determine the relative severity or grade of laryngitis due to the absence of objective testing such as formal virological identification or laryngoscopic features. Third, our study detected small effects as statistically significant. For example, the odds ratio for HZI was 1.05 for inflammatory disease. A 5% risk can be a statistically significant result that is also clinically irrelevant. Finally, our study sample (e.g., patients under 20 years old were excluded) limits the generalizability of our conclusions because HZI risk increases with age. Further prospective cohort studies with more detailed diagnostic tests such as serologic tests or laryngoscopy are needed to examine herpes zoster laryngitis.

In conclusion, in the herpes zoster group compared with the control group, inflammatory diseases increased by 5% and reflux diseases increased by 20%. The control group was matched and adjusted for demographic factors and previous medical histories of hypertension, diabetes, dyslipidemia, ischemic heart disease, cerebral stroke, and depression histories.
